# Postprandial NMR-Based Metabolic Exchanges Reflect Impaired Phenotypic Flexibility across Splanchnic Organs in the Obese Yucatan Mini-Pig

**DOI:** 10.3390/nu12082442

**Published:** 2020-08-14

**Authors:** Marie Tremblay-Franco, Nathalie Poupin, Aurélien Amiel, Cécile Canlet, Didier Rémond, Laurent Debrauwer, Dominique Dardevet, Fabien Jourdan, Isabelle Savary-Auzeloux, Sergio Polakof

**Affiliations:** 1 Toxalim (Research Centre in Food Toxicology), Université de Toulouse, 31300 Toulouse, France; marie.tremblay-franco@inrae.fr (M.T.-F.); nathalie.poupin@inrae.fr (N.P.); aurelien.amiel@inrae.fr (A.A.); cecile.canlet@inrae.fr (C.C.); laurent.debrauwer@inrae.fr (L.D.); Fabien.Jourdan@inrae.fr (F.J.); 2 Axiom Platform, MetaToul-MetaboHUB, National Infrastructure for Metabolomics and Fluxomics, 31300 Toulouse, France; 3 INRAE, Unité de Nutrition Humaine, Université Clermont Auvergne, 63000 Clermont-Ferrand, France; didier.remond@inrae.fr (D.R.); dominique.dardevet@inrae.fr (D.D.); isabelle.savary-auzeloux@inrae.fr (I.S.-A.)

**Keywords:** arteriovenous differences, postprandial, high fat-high sucrose diet, mini-pigs, energy metabolism, metabolomics, liver, intestine, phenotypic flexibility

## Abstract

The postprandial period represents one of the most challenging phenomena in whole-body metabolism, and it can be used as a unique window to evaluate the phenotypic flexibility of an individual in response to a given meal, which can be done by measuring the resilience of the metabolome. However, this exploration of the metabolism has never been applied to the arteriovenous (AV) exploration of organs metabolism. Here, we applied an AV metabolomics strategy to evaluate the postprandial flexibility across the liver and the intestine of mini-pigs subjected to a high fat–high sucrose (HFHS) diet for 2 months. We identified for the first time a postprandial signature associated to the insulin resistance and obesity outcomes, and we showed that the splanchnic postprandial metabolome was considerably affected by the meal and the obesity condition. Most of the changes induced by obesity were observed in the exchanges across the liver, where the metabolism was reorganized to maintain whole body glucose homeostasis by routing glucose formed de novo from a large variety of substrates into glycogen. Furthermore, metabolites related to lipid handling and energy metabolism showed a blunted postprandial response in the obese animals across organs. Finally, some of our results reflect a loss of flexibility in response to the HFHS meal challenge in unsuspected metabolic pathways that must be further explored as potential new events involved in early obesity and the onset of insulin resistance.

## 1. Introduction

The postprandial period represents one of the most challenging phenomena in whole-body metabolism, given that following meal intake, body metabolism must adapt to major changes in the blood composition of nutrients. Interestingly, this adaptation could be used as an indicator of adapted or altered response to a given nutritional challenge, which is named today as the “phenotypic flexibility”, and relates to the multiple processes in the (molecular) physiology involved in maintaining this metabolic resilience [1]. Whereas during healthy physiological conditions, this flexibility will be able to handle the energy and nutrients overload resulting from a single and acute high fat meal, the adaptive processes guaranteeing homeostasis might reach their limits if exposure to such a diet becomes chronic. It is as yet unclear at which stage during overfeeding these adaptive processes can be regarded as part of normal metabolic flexibility or whether they reflects negative side effects in the spectrum of the metabolic syndrome [2]. Thus, we have recently shown that in mini-pigs overfed with a high fat–high sucrose (HFHS) diet for two months, major modifications in the postprandial metabolism occur after only one week of feeding [3,4,5,6].

In the last decade, metabolomics has proved to be a valuable tool for gaining better understanding of metabolic processes [7,8] and to be particularly adapted for studying the time-course adaptations of the metabolism to obesogenic diets, as shown by us and others [3,9,10,11,12]. Beyond the fasting condition, measuring the postprandial metabolome in response to a given meal (such as an HFHS meal), is of prime importance since human beings spend most of their time in the postprandial state [13]. Interestingly, it has been shown that during the postprandial period the metabolome is highly responsive and flexible [14] and that the number of metabolites signing a particular pathophysiological condition was significantly increased with respect to the fasting condition [15].

However, so far, the majority of untargeted metabolomics studies has focused on discovering biomarkers by profiling blood or urine samples [12,16], which provides an interesting static snapshot of the whole body metabolism but offers limited mechanistic information to which tissues/organs respond differentially [17]. Only by determining the arteriovenous (AV) concentration of metabolites across a given organ is it possible to know what is up-taken or released, and then estimate the metabolic modifications of the organ explored. AV analysis of the metabolome is therefore a particularly relevant approach to better understand the metabolism of a given organ by extending the exploration to not only a few, but rather hundreds of metabolites, as demonstrated in healthy humans [17] and pigs [18]. We recently explored this concept further, by applying this strategy across the liver and the intestine of obese insulin resistant (HFHS-fed) mini-pigs [19]. However, this study was performed in the postabsorptive period (overnight fasting) only, when homeostasis had most likely returned to steady state following the last HFHS meal.

To reveal other metabolic adaptations and have a better overview of modifications occurring outside the fasting state, in the current study we extended the metabolomics AV strategy to the postprandial period, during which more sensitive changes in the metabolic resilience have been demonstrated [20]. We used the multi-catheterized mini-pig model of obesity and insulin resistance to apply a metabolomics approach (NMR-based metabolomics platform) allowing the multi-organ and high-throughput exploration of the metabolism [19].

## 2. Materials and Methods

### 2.1. Animals and Experimental Procedure

The study involved five female adult Yucatan mini-pigs (31.5 ± 1.4 kg). Three weeks before the experiment, the mini-pigs were surgically fitted with a catheter in the abdominal aorta artery (Art), the portal vein (PV), and the hepatic vein (HV). They were housed in subject pens in a ventilated room with controlled temperature (21 °C) and a regular light cycle (L12:D12). They were fed once daily with 400 g/d of a concentrated feed containing 17.5% protein, 3.2% fat, 4.3% cellulose, and 5.2% ash (Porcyprima; Sanders Centre Auvergne, Aigueperse, France), and had free access to tap water. All the procedures were performed in accordance with the guidelines formulated by the European Community for the use of experimental animals (L358-86/609/EEC, Council Directive, 1986). The protocol was approved by the Ethical Committee for Animal Experimentation-Auvergne, authorization 02090.01.

After the recovery period on the regular diet, mini-pigs were fed an HFHS diet consisting of a regular pig diet enriched with fat (12% palm oil) and sugar (10% sucrose) (1 kg/day, 13.3 kJ/day) for two months. Then, animals ingested the whole mixture in no more than 10 min. After overnight fasting, blood was sampled through the three catheters simultaneously in heparinized tubes, on the first (d0) and last (60 days) HFHS meals. Blood was also collected during the postprandial period: 60, 180, 330 and 510 min after the meal. Blood was centrifuged at 4500× *g* for 10 min, with the plasma rapidly collected and stored at −80 °C until further analyses. The homeostatic model assessment (HOMA)2-IR, was calculated by the program HOMA Calculator v2.2.3 (http://www.dtu.ox.ac.uk/ToolsSoftware/; Oxford, UK).

### 2.2. Plasma Metabolomics

Two-hundred microliters of plasma samples was mixed with 500 µL of phosphate buffer (pH 7.0) prepared in deuterated water, and then centrifuged at 5500× *g* at 4 °C for 15 min, after which 600 µL of supernatant was transferred to 5 mm NMR tubes. All 1H NMR spectra were obtained on a Bruker DRX-600-Avance III HD NMR spectrometer operating at 600.13 MHz for 1H resonance frequency using an inverse detection 5 mm 1H-13C-15N-31P cryoprobe attached to a Cryoplatform (the preamplifier unit). The 1H NMR spectra were acquired at 300 K using the Carr–Purcell–Meiboom–Gill (CPMG) spin-echo pulse sequence with presaturation, with a total spin-echo delay (2nτ) of 64 ms to attenuate broad signals from proteins and lipoproteins. A total of 256 transients were collected in 32 K data points using a spectral width of 20 ppm, a relaxation delay of 2 s, and an acquisition time of 1.36 s. Prior to Fourier transformation, an exponential line broadening function of 0.3 Hz was applied to the FID. All NMR spectra were phased and baseline-corrected; then, the data were reduced using AMIX (version 3.9 Bruker, Rheinstetten, Germany) to integrate 0.01 ppm wide regions corresponding to the δ 8.5−0.5 ppm region. The δ 5.1−4.5 ppm region, which includes the residual water resonance, was excluded. A total of 594 NMR buckets were included in the data matrix. To account for differences in sample concentration, each integrated region was normalized to the total spectral area.

### 2.3. Statistical Analyses

Multivariate analyses were used to study the effect of the HFHS diet along time course on the metabolome. Principal components analysis (PCA) was performed first to reveal intrinsic clusters and detect possible outliers. Partial least squares-discriminant analysis (PLS-DA) was then used to model the relationship between group and spectral data. PLS-DA is similar to PCA but uses discriminant variables that correlate to class membership. Before analysis, orthogonal signal correction (OSC) filtering was used to remove variability not linked to the conditions studied (physiological, experimental or instrumental variation). Filtered data were mean-centered and Pareto scaled. For all the figures, Hotelling’s T2 statistics were used to construct 95% confidence ellipses. The R2Y parameter represents the explained variance. Sevenfold cross-validation was used to determine the number of latent variables to include in the PLS-DA model and to estimate the predictive ability (Q^2^ parameter) of the adjusted model. In addition, the robustness and validity of the PLS-DA models were calculated using a permutation test (number of permutations = 200). The Variable Importance in the Projection (VIP > 0.8) and the Kruskal–Wallis test with False Discovery Rate multiple testing correction [21] (corrected *p* < 0.05) were used to select discriminant and significant NMR buckets.

To take into account repeated data (samples collected from different vessels at different postprandial time points on the same individuals), the multilevel approach [22] was applied beforehand to split the within-subject variation from to between-subject variation. Postprandial time and vessel factors were included in the design. Sparse Partial Least Squares—Discriminant analysis (sPLS-DA [23]) was then run on the within matrix. The PLS-DA method seeks linear combinations of NMR buckets that best separate groups (postprandial time and vessel in this study). The sparse version of PLS-DA uses L1 constraints when estimating the weights of linear combinations to perform variable selection. The optimal number of latent variables to include in the PLS-DA model and the number of variables to select on each dimension (PLS latent variable) were chosen using cross-validation. The Area Under the Curve (AUC) criterion was used to assess model quality. Permutation tests (100 iterations) were performed to evaluate model robustness. The Variable Importance in the Projection (VIP > 0.8) was used to identify discriminant NMR buckets. Finally, significant buckets were selected using the Kruskal–Wallis test, with False Discovery Rate multiple testing correction. The R mixOmics package [24] was used to perform these analyses.

### 2.4. Identification of Metabolites Exchanges

For the exchange of metabolites across the organs we calculated the ratios between the artery and the veins as in [19]: Art/PV for the intestine, and ((Art*0.2) + (PV*0.8))/HV for the liver [19,25]. We then expressed these ratios as the percentage of induction from the steady state, considering the steady state as inflow/outflow = 1. Thus, metabolites with positive values are considered as taken up (as a net balance), while those with negative values are considered as released by a given organ,

(1) $${\%\mathrm{induction}}_{\left(\mathrm{met}\;m,\;\mathrm{indiv}\;i,\;\mathrm{day}\;d\right)}=\left(\frac{{\mathrm{Art}}_{\mathrm{met}\;m,\;\mathrm{indiv}\;i,\;\mathrm{day}\;d}}{{\mathrm{PV}}_{\mathrm{met}\;m,\;\mathrm{indiv}\;i,\;\mathrm{day}\;d}}-1\right)\times 100,\;\mathrm{for}\;\mathrm{intestine}$$

(2) $${\%\mathrm{induction}}_{\left(\mathrm{met}\;m,\;\mathrm{indiv}\;i,\;\mathrm{day}\;d\right)}=\left(\frac{{\mathrm{Art}}_{\mathrm{met}\;m,\;\mathrm{indiv}\;i,\;\mathrm{day}\;d}\times 0.2+{\mathrm{PV}}_{\mathrm{met}\;m,\;\mathrm{indiv}\;i,\;\mathrm{day}\;d}\times 0.8}{{\mathrm{HV}}_{\mathrm{met}\;m,\;\mathrm{indiv}\;i,\;\mathrm{day}\;d}}-1\right)\times 100,\;\mathrm{for}\;\mathrm{liver}$$

where Art, PV, and HV are the NMR integration area of each metabolite measured in plasma samples from Art, PV, and HV, respectively. For each metabolite, a signal without overlapping was chosen for the integration ratio calculation. In order to determine if the exchange was significant at each time point (T0, T60, T180, T330, and T510), the arterial and venous blood values were compared using a paired-*t*-test SigmaPlot v12.3 (Systat Software, San Jose, CA, USA).

Data from each vessel were also analyzed to determine the exchanges during the whole postprandial period for each metabolite by calculating the AUC using the trapezoid method. Then, the exchange (% of induction) was calculated as above, using the AUC values. In order to determine if the exchange was significant during the postprandial period overall, the AUC values for the incoming and outgoing blood were compared using a paired-t-test. To determine if the exchange of a metabolite was affected by the meal intake, the AUC value was divided by postprandial period of time (510 min), and compared to the fasting value using a paired-*t*-test. Finally, the specific effect of the meal intake was evaluated by calculating the delta change between the maximum and fasting exchange values for a given metabolite. Comparisons of the delta changes between D0 and D60 were performed using a paired-*t*-test.

In order to explore the impact of the meal, sampling site and obesity condition, those metabolic features with at least one postprandial point significantly different from the fasting value were subjected to a hierarchical clustering analysis (HCA) using the MetaboAnalyst module. Auto-scaling was carried out and Euclidian distance and the Ward aggregation criterion were used [26]. In order to determine the connection between the postprandial response and the health outcomes, correlations were performed between the postprandial AUC of all the annotated metabolites and the HOMA-IR (marker of insulin resistance) or the BW (marker of weight gain/obesity) using the statistical module of MetaboAnalyst (Pearson correlation).

## 3. Results

After two months of HFHS feeding the mini-pigs developed an obesity and insulin resistance-like phenotype, with a significant increase in HOMA-IR index (from 0.075 ± 0.03 to 0.41 ± 0.08) and body weight (from 31.5 ± 1.4 kg to 44.7 ± 1.7 kg), most likely as the consequence of fat deposition in the visceral and subcutaneous adipose tissue [3]. More details about biochemical and clinical phenotyping were published previously [5,6]. The current study is exclusively based on the analyses of the postprandial response. Fasting data were presented in a previous publication [19].

**Figure 1:**
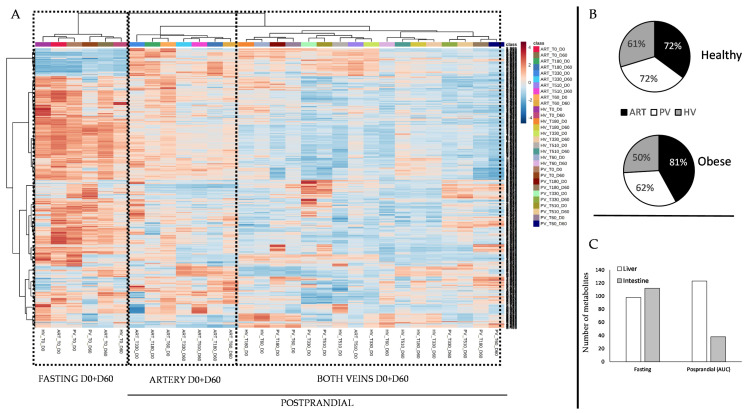
(**A**) Heatmap with hierarchical clustering (Euclidian distance and ward aggregation) of significant metabolic features (at least one point different from the fasting condition). Lowest ion intensity is shown in bleu while maximum intensity is shown in red. ART, artery; PV, portal vein; HV, hepatic vein. (**B**) percentage of metabolites postprandially different (at least one postprandial point different from fasting) between the healthy and the obese animals. (**C**) Number of metabolites differentially exchanged across the liver and the intestine between the obese and healthy animals in the fasting and postprandial states (Area Under the Curve (AUC)).

The first approach to evaluate the differences between the different sampling sites was to perform a clustering analysis of the metabolic features for which at least one time point after the meal was different from the fasting condition (Figure 1A). We observed that two main clusters appeared, mainly separating nutritional statuses: one of them included the pre-meal time and the other included all the postprandial times. Interestingly, inside the pre-meal cluster, the healthy and the obese conditions were separated. However, inside the postprandial cluster, the main classifications corresponded to the artery vs. veins (HV + PV) separation, irrespectively of the healthy/obese condition. The impact of the meal on the metabolome was considerable: in the healthy condition 72% of the metabolic features detected were altered by the meal at the artery (81% in the obese animals) and PV (62% in the obese animals) levels, and 61% at the HV level (50% in the obese animals) (Figure 1B). When looking at the exchanges, we observed that the liver exchanges were much more affected than those across the intestine (Figure 1C). In the fasting state, the number of metabolic features differentially exchanged between the healthy and the obese conditions was similar: 98 at the level of the liver and 112 across the intestine. At the postprandial level, the picture was rather different: when considering the total AUC 123 metabolites were differentially exchanged across the liver, and only 38 across the intestine.

**Figure 2:**
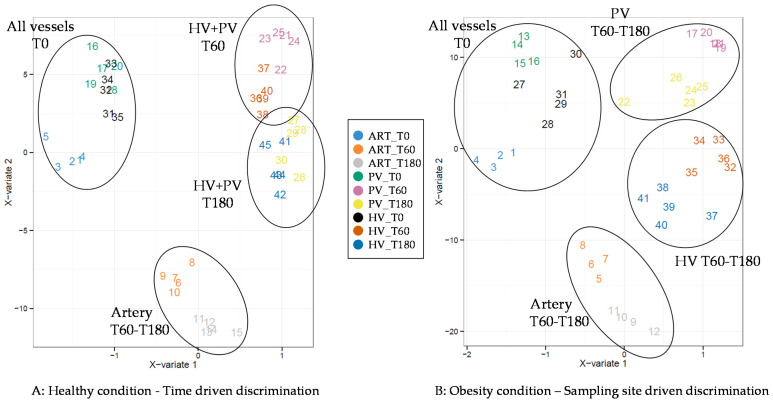
Scores plot of multi-level sparse partial least squares discriminant analysis model (*n* = 5) after OSC filter. Analyses were done at the healthy (**A**) and obese (**B**) conditions. Analyses were focused on the T0, T60, and T180 min. ART, artery; PV, portal vein; HV, hepatic vein.

The main objective of the present study was to explore the differences induced by the obesity condition on the different metabolomes as a function of the sampling site and exchanges. The first approach was performed on individual vessels and days, which confirmed the major impact of the fasted-to-fed transition (Figure S1 (Supplementary Materials)). In order to analyze both, the postprandial time and sampling site, we then applied multi-level sPLS-DA analyses to our data (Figure 2). We focused on the first sampling times after the meals (0–180 min), as they concentrated most of the altered metabolites between the healthy and the obese condition. Model validation is provided in Supplementary Figure S2 (Supplementary Materials). As for the clustering, the supervised analysis showed that in the healthy animals, the main discrimination was driven by the nutritional condition (before and after the meal), while the second component separated the artery metabolome from those of the veins. Interestingly, we also observed that for each vessel, the postprandial organization of the groups was driven by the time after the meal: thus, for the artery the 60 min and 180 min were grouped together (and separated from the 0 min), but this was not the case for the veins. Both the PV and HV were classified together by time: a 60 min cluster and a 180 min cluster. The situation in the obese animals was not the same: although the first axis also discriminated the pre- and post-meal measures, the postprandial classification was not made on the same basis as in the healthy condition. Whereas the artery (representing the general circulation) did not change from the healthy condition (60 min and 180 min together), the rest of the classification was driven by the vessels and not time: the HV and PV were then separated, with the 60 min and 180 min time points together for each of them.

**Figure 3:**
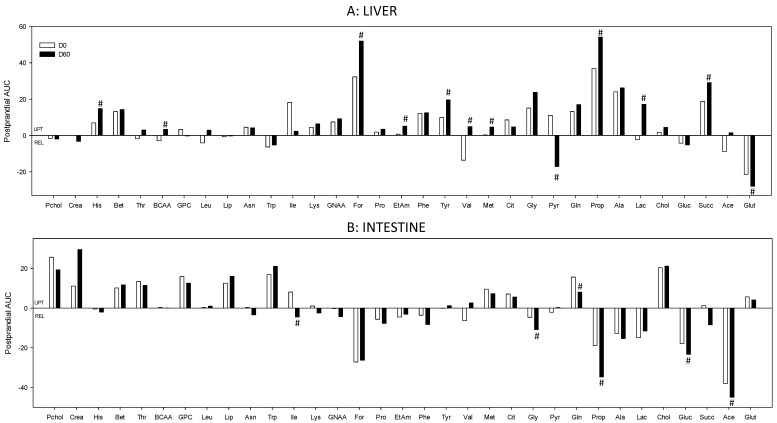
Arteriovenous global exchanges during the postprandial period (AUC) across the liver (**A**) and the intestine (**B**). Data is presented in % of induction (mean): positive values = taken up by the organ; negative values = released by the organ. Data were analyzed using a paired *t*-student test. #, significantly different from D0, *p* < 0.05. Detailed data are presented in the supplemental Tables S1 and S2 (Supplementary Materials). Pchol, phosphocholine; Crea, creatine; His, histidine; Bet, betaine; Thr, threonine; BCAA, branched-chain amino acids; GPC, glycerophosphocholine; Leu, leucine; Lip, lipids; Asn, asparagine; Trp, tryptophan; Ile, isoleucine; Lys, lysine; GNAA, gluconeogenic amino acids; For, formic acid; Pro, proline; EtAm, ethanolamine; Phe, phenylalanine; Tyr, tyrosine; Val, valine; Met, methionine; Cit, citrate; Gly, glycine; Pry, pyruvate; Gln, glutamine; Prop, propionate; Ala, alanine; Lac, lactate; Chol, choline; Gluc, glucose; Succ, succinate; Ace, acetate; Glut, glutamate.

**Figure 4:**
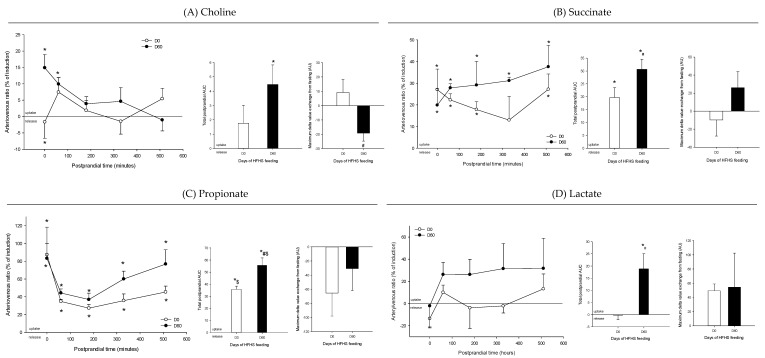
Arteriovenous fold change (A/V) of (**A**) choline, (**B**) succinate, (**C**) propionate, and (**D**) lactate across the liver on five Yucatan mini-pigs submitted to a HFHS diet during two months. Data is presented in % of induction (mean + sem) and the exchange was analyzed using a repeated-measures *t*-student test. *, significantly different from 0 (equilibrium), *p* < 0.05. For the A/V ratios and total postprandial AUC, positive values = taken up by the organ; negative values = released by the organ. #, significantly different from D0, *p* < 0.05; $, significantly different from the fasting condition, *p* < 0.05. The maximum delta value exchange from fasting is also presented: the positive and negative values represent an increase or decrease exchange following the meal, respectively.

**Figure 5:**
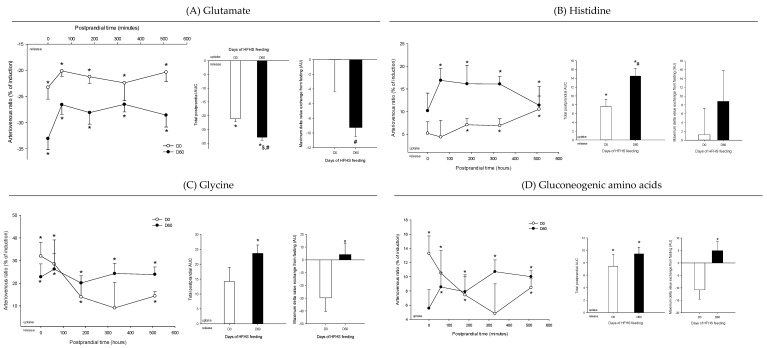
Arteriovenous fold-change (A/V) of (**A**) glutamate, (**B**) histidine, (**C**) glycine, and (**D**) gluconeogenic amino acids across the liver on five Yucatan mini-pigs submitted to a HFHS diet during two months. Data is presented in % of induction (mean + sem) and the exchange was analyzed using a repeated-measures *t*-student test. *, significantly different from 0 (equilibrium), *p* < 0.05. For the A/V ratios and total postprandial AUC, positive values = taken up by the organ; negative values = released by the organ. #, significantly different from D0, *p* < 0.05; $, significantly different from the fasting condition, *p* < 0.05. The maximum delta value exchange from fasting is also presented: the positive and negative values represent an increase or decrease exchange following the meal, respectively.

**Figure 6:**
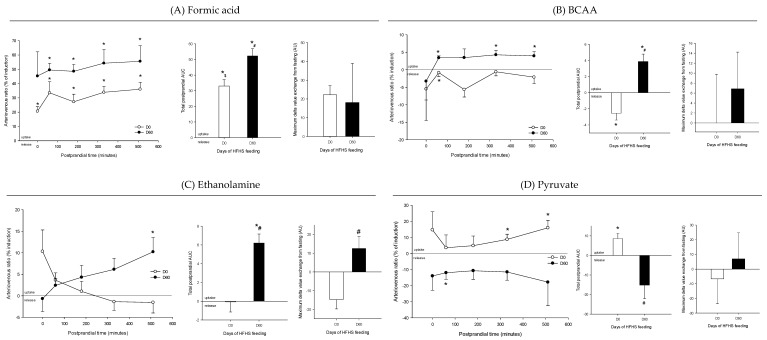
Arteriovenous fold change (A/V) of (**A**) formic acid, (**B**) branched-chain amino acids (BCAA), (**C**) ethanolamine, and (**D**) pyruvate across the liver on five Yucatan mini-pigs submitted to a HFHS diet during two months. Data is presented in % of induction (mean + sem) and the exchange was analyzed using a repeated-measures *t*-student test. *, significantly different from 0 (equilibrium), *p* < 0.05. For the A/V ratios and total postprandial AUC, positive values = taken up by the organ; negative values = released by the organ. #, significantly different from D0, *p* < 0.05; $, significantly different from the fasting condition, *p* < 0.05. The maximum delta value exchange from fasting is also presented: the positive and negative values represent an increase or decrease exchange following the meal respectively.

Figure 3A, Figure 4, Figure 5 and Figure 6 and Table 1 summarize the postprandial changes observed in the annotated metabolites across the liver. Those metabolites showing significant differential changes between the healthy and the obese conditions during the postprandial period were chosen to illustrate the discussion. More details are provided in Table S1 (Supplementary Materials).

The total AUC of the % of induction (AV ratios) provides valuable integrated information about the exchange of a given metabolite during the whole postprandial period (from 0 to 510 min). At the hepatic level, in the healthy condition 12 of the 32 annotated metabolites were up-taken in the postprandial state, while only four (glutamate, tryptophan, glucose, and valine) were actually released by the liver (Figure 3A). For two of them, the exchange was observed only following the meal (exchange different from fasting): higher postprandial uptake for formic acid and lower release for propionate, when compared with the fasting condition. When compared to the healthy condition, several metabolites showed an increased exchange at D60, including succinate, propionate, glutamate, histidine, formic acid, and tyrosine, or were additionally exchanged at D60 whereas they were not at D0 (ethanolamine, BCAA = branched chain amino acids, lactate). Conversely, only two metabolites (isoleucine and valine) displayed decreased exchange and were no longer significantly exchanged by the end of the trial. Interestingly, pyruvate showed a shifted pattern in its exchange between D0 and D60, being taken up at D0 and released at D60. Finally, the maximum delta value exchange from fasting provided particularly interesting information about the postprandial exchange induction (positive or negative) following the meal. The only metabolite that showed a negative induction was choline: when compared to the healthy situation, its uptake was significantly reduced by the meal. For the other altered metabolites, including gluconeogenic AA (alanine, glutamine, glutamate, glycine, and threonine), methionine, and ethanolamine, the delta value of exchange was rather positively affected by the meal. More details are provided on the Table S1 (Supplementary Materials).

**Figure 7:**
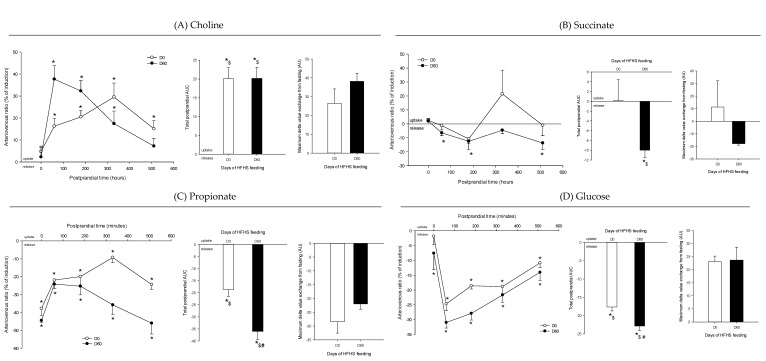
Arteriovenous fold-change (A/V) of (**A**) choline, (**B**) succinate, (**C**) propionate, and (**D**) glucose across the intestine on five Yucatan mini-pigs submitted to a HFHS diet during two months. Data is presented in % of induction (mean + sem) and the exchange was analyzed using a repeated-measures *t*-student test. *, significantly different from 0 (equilibrium), *p* < 0.05. For the A/V ratios and total postprandial AUC, positive values = taken up by the organ; negative values = released by the organ. #, significantly different from D0, *p* < 0.05; $, significantly different from the fasting condition, *p* < 0.05. The maximum delta value exchange from fasting is also presented: the positive and negative values represent an increase or decrease exchange following the meal, respectively.

**Figure 8:**
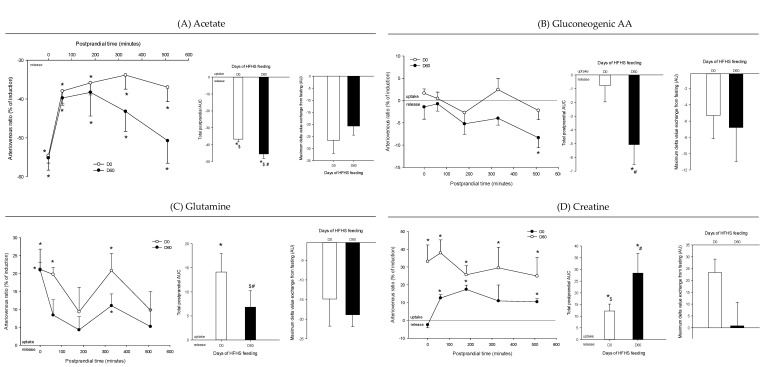
Arteriovenous fold change (A/V) of (**A**) acetate, (**B**) gluconeogenic amino acids, (**C**) glutamine, and (**D**) creatine across the intestine on five Yucatan mini-pigs submitted to a HFHS diet during two months. Data is presented in % of induction (mean + sem) and the exchange was analyzed using a repeated-measures *t*-student test. *, significantly different from 0 (equilibrium), *p* < 0.05. For the A/V ratios and total postprandial AUC, positive values = taken up by the organ; negative values = released by the organ. #, significantly different from D0, *p* < 0.05; $, significantly different from the fasting condition, *p* < 0.05. The maximum delta value exchange from fasting is also presented: the positive and negative values represent an increase or decrease exchange following the meal respectively.

**Figure 9:**
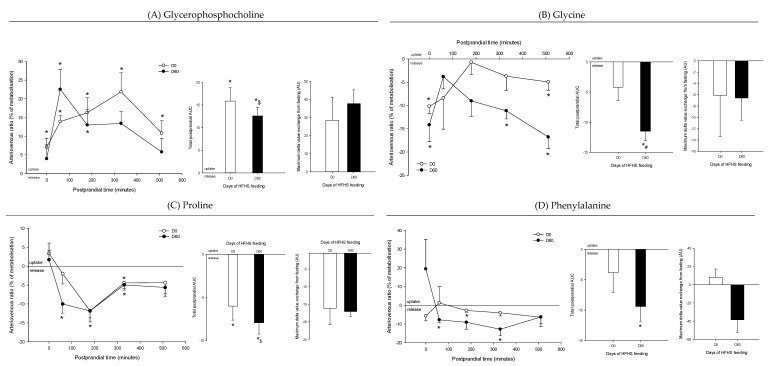
Arteriovenous fold change (A/V) of (**A**) glycerophosphocholine, (**B**) glycine, (**C**) proline, and (**D**) phenylalanine across the intestine on five Yucatan mini-pigs submitted to a HFHS diet during two months. Data is presented in % of induction (mean + sem) and the exchange was analyzed using a repeated-measures t-student test. *, significantly different from 0 (equilibrium), *p* < 0.05. For the A/V ratios and total postprandial AUC, positive values = taken up by the organ; negative values = released by the organ. #, significantly different from D0, *p* < 0.05; $, significantly different from the fasting condition, *p* < 0.05. The maximum delta value exchange from fasting is also presented: the positive and negative values represent an increase or decrease exchange following the meal, respectively.

Figure 3B, Figure 7, Figure 8 and Figure 9 and Table 2 summarize the postprandial changes observed in the annotated metabolites across the intestine, and display the metabolites showing significant differential changes between the healthy and the obese conditions during the postprandial period. The profile of exchange at the intestine level was similar to that of the liver: most of the annotated metabolites (11 of 33) were taken up during the postprandial period, while seven of them were actually released (Figure 3B). Among the metabolites exchanged during the postprandial period, propionate, alanine, glucose, and acetate were released and choline, creatine, betaine, and lipids were taken up only during the postprandial period.

The obesity condition considerably affected the intestinal exchanges of metabolites (Table S2 (Supplementary Materials)). Thus, more propionate, glucose, and acetate were released in the obese animals. Interestingly, succinate, glycine, and gluconeogenic AA were also released in the obese animals, while during the healthy condition their exchanges were neutral. On the other hand, creatine was more actively taken up in the obese animals, while glutamine showed the inverse profile.

**Figure 10:**
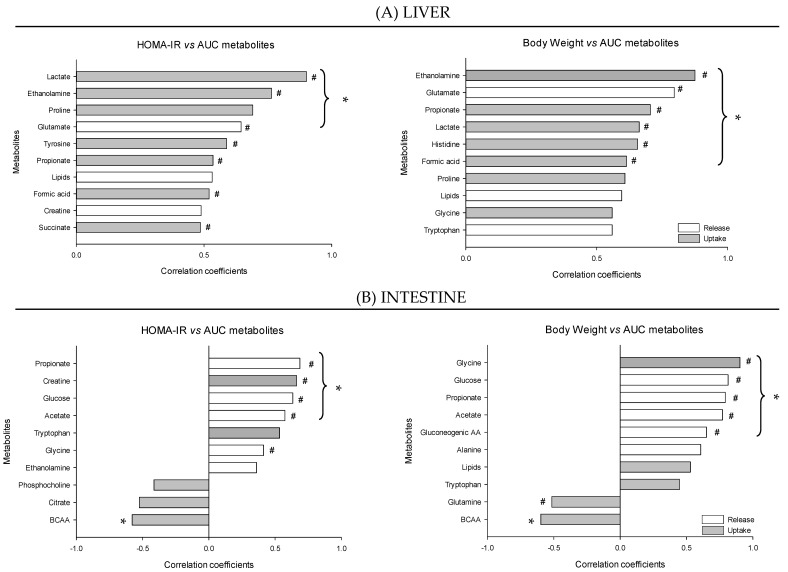
Pearson correlations performed at the liver (**A**) or intestine (**B**) level between the HOMA-IR or the BW and the AUC exchange values for each metabolite to identify metabolites that are release or take up in greater/smaller amount depending on the HOMA-IR or BW. Correlations were computed using the absolute values of the exchange AUC at both D0 and D60 in order to consider in a similar way the release (negative AUC values) and uptake (positive AUC values) exchanges. Metabolites for which the absolute value of the AUC did not reflect the change in the exchange between D0 and D60 because the AUC value was switching sign (i.e., switching from uptake to release or from release to uptake) between D0 and D60 were excluded from the analysis. *, significant correlation between a given metabolite and health outcome (HOMA-IR or obesity), *p* < 0.05; #, AUC significantly different from D0, *p* < 0.05. The figures are showing the top10 metabolites. See the supplemental Tables S3–S6 (Supplementary Materials) for more details.

Figure 10 shows the top ten correlations performed between the two markers of obesity and insulin resistance and the postprandial integrated AUC of metabolite exchanges for the liver and the intestine. Insulin resistance onset was correlated positively with the postprandial AUC of several metabolites, including lactate, ethanolamine and proline (up-taken) and glutamate (released) across the liver and propionate, glucose and acetate (released), and creatine (up-taken) across the intestine. HOMA-IR correlated also negatively with the BCAA uptake across the intestine. Interestingly, for most of them (except BCAA and proline) a difference in the AUC between the healthy and the obese/insulin resistance condition was also observed. Some of the metabolites correlated with the HOMA-IR index were also associated with the weight gain, including ethanolamine, glutamate, and lactate across the liver and glucose propionate and acetate across the intestine. Other correlated metabolites included up-taken propionate, histidine, and formic acid in the liver, and up-taken glycine and released gluconeogenic AA across the intestine. See the supplemental Tables S3–S6 (Supplementary Materials) for more details.

## 4. Discussion

Previous studies have shown that a metabolomics exploration following a standardized meal test is more informative on metabolic status and subtle health effects than the quantification of the homeostatic (fasting) situation [15,27]. However, those studies were limited to the general blood circulation, which provides little information about the metabolism of specific tissues/organs. Here, we showed for the first time that the postprandial metabolome across the splanchnic area is highly modified as a consequence of food absorption and tissue metabolism. Some of the altered metabolites were strongly correlated to the HOMA-IR index and weight gain, and are discussed in the context of the splanchnic metabolism adaptation to the HFHS diet, and as part of a postprandial signature related to the insulin resistance and obesity onset.

### 4.1. The Metabolomes of the Splanchnic Area Are Greatly Altered Following Meals

In accordance with our previous study on general circulation in healthy mini-pigs [12], we confirm that the nutritional status of the animals was a major perturbation for individual metabolic homeostasis. Our current data allow further extending this idea specifically to the splanchnic area. Overall, more than 50% of the metabolome detected on each vessel was actually impacted by the meal intake. Interestingly, fasting vs. postprandial discrimination was less clear at D60, suggesting that the obese animals were metabolically in a permanent postprandial state, and that their capacity to respond to the meal challenge have been compromised [1], as it occurred at the onset of the insulin resistance [28].

Several observations of the global metabolomes at the different sampling sites also pointed to specific metabolic modifications of the splanchnic organs. First, in the hierarchical classification the postprandial cluster containing the arteries and representing the general circulation was classified apart from that containing the veins together. In addition, inside the veins cluster, the metabolomes from the healthy and obese animals were further separated, most likely due to the obesity condition, although a contribution of the digestive tract’s adaptation to the new diet cannot be ruled out [29]. Second, we showed that while the changes observed in the healthy animals were driven by the postprandial time, those recorded in the obese animals were more driven by the sampling-site, suggesting a metabolic overload of the splanchnic organs during the weight gain period. Furthermore, particularly for the liver, the analysis of exchanges following the meal (AUC) revealed that the postprandial period was more sensitive in discriminating the obese vs. healthy conditions than the fasting status. Therefore, we address here the first data concerning the postprandial alterations in the metabolome exchanged across splanchnic organs.

### 4.2. Adaptive Changes Take Place in the Splanchnic Metabolism to Maintain Postprandial Glucose Homeostasis at the Onset of Obesity

At obesity onset, the whole body metabolism must adapt to maintain glucose homeostasis and delay diabetes occurrence. Recent studies have documented the importance of postprandial hyperglycemia as a risk factor for all-cause and cardiovascular mortality in the normal population and the fact that postprandial insulin resistance may be an early stage of the process of diabetes mellitus [30,31]. We observed that several metabolic adaptations linked to the glucose–amino acid metabolic cross-road occurred to maintain glucose production under control following the HFHS meal, and this way better control glycaemia. Thus, as expected in the healthy animals, the gluconeogenic AA uptake by the liver was inhibited (yet still present as shown by previous studies on pigs [32]) following the meal: a mechanism that was no longer observed when these same animals became obese. Interestingly, these AA were indeed taken up, as the latter occurred in the insulin resistant state [33] and in line with the concomitant larger release of gluconeogenic AA by the intestine. Other contributors to hepatic glucose production, like lactate and the gut microbiote-derived metabolites (propionate and succinate) were also taken up more considerably following the meal in the obese mini-pigs. This is in agreement with the altered levels of lactate in diabetic patients [34,35] and consistent with the propionate and succinate intestinal production shown at the beginning of the postabsorptive period [36,37]. Interestingly, lactate uptake was also strongly correlated with HOMA-IR and weight gain, suggesting that its altered postprandial exchange can be connected to the insulin resistance and obesity onset. It is noteworthy that, despite the increased uptake of numerous substrates potentially able to boost postprandial hepatic glucose production and potential hyperglycaemia (a common feature of postprandial insulin resistance [38]), the obese animals did not exhibit greater postprandial hepatic glucose export [4]. We hypothesized that the higher glucose production would therefore be stored in the liver though the “indirect or paradoxical” [39] and the glyconeogenenic [40] pathways that convert lactate and gluconeogenic AA directly into glycogen. The consequence of this adapted “metabolic strategy” was not only the storage of the glucose produced postprandially for use during the postabsorptive period, but also the attenuation of hepatic glucose output, preventing postprandial glucose excursion [4]. This global picture of the intermediate hepatic metabolism was completed by a shift in substrate utilization for energy purposes, in which hepatic metabolism was further supported by the dietary lipids, displacing dietary glucose utilization and explaining the leaking of the pyruvate observed [41].

### 4.3. The Loss of Postprandial Flexibility in the Splanchnic Organs as a Symptom of Early Metabolic Alterations

In our study, the HFHS meal acted as a challenge test, so subtle differences established during obesity and insulin resistance onset were more visible during the postprandial period, including some blunted responses. Several metabolite exchanges across the liver showed specific postprandial changes in the obese condition related to lipid and purine metabolisms that were compatible with the onset of insulin resistance [42]. In the case of ethanolamine (highly correlated with HOMA-IR and weight gain), its increased postprandial uptake in the obese animals could be related to an adaptive response to enhance the capacity of the liver to handle the dietary lipids after the meal. However, this does not agree with the postprandial uptake of choline (also essential to phospholipid synthesis), which was rather reduced in the obese animals, therefore limiting the potential of the pathway. Thus, although some aspects of the lipid handling capacities seemed to remain flexible enough to adapt to the metabolic needs imposed by the HFHS meal, others were not, which could eventually lead to hepatic lipid accumulation [43], one of the driving features of hepatic insulin resistance [42]. The liver of the obese animals also showed altered formic acid exchanges. It is known that increased purine synthesis can stimulate the formic acid uptake to sustain pathway potential [44], which could explain the origin the low levels of formic acid recently reported in obese patients [45], and is coherent with the positive correlation observed with the weight gain in our study.

At the intestinal level, the obese animals lost their postprandial induction of creatine uptake, and remained with a high uptake level even during the postabsorptive state. This could be due to the enhanced release by other organs and linked to the high circulating creatine levels reported in obese subjects [46] and rodent models of insulin resistance [47], and is supported by the correlation found with the HOMA-IR in our study. On the other hand, we observed that the intestinal utilization of glycine in the healthy individuals was compromised in the obese condition. Thus, permanent glycine release by the intestine exposed the liver to high levels of this AA, leading to its enhanced hepatic uptake to maintain whole body glycine homeostasis. In this sense, the increased enterohepatic cycle of bile salts (conjugated to taurine and glycine) as part of an adaptive mechanism to improve lipid absorption could be considered [48], in line with the dysregulation of intestinal lipid metabolism observed in insulin resistant rodents [49] and humans [50].

### 4.4. The Postprandial Metabolism of the Splanchnic Organs Adapts to Circulating Nutrient Availability

Some of the results observed were also related to an adjustment of the metabolism to face the increased dietary availability of sugar and fat. Thus, based on the increased postprandial release of glutamate by the liver in the obese animals, we show for the first time that the nitrogen sparing mechanism resulting from HFHS feeding observed in the fasting state [19,51], was also functional during the postprandial period. The BCAA situation also showed major changes in the AV exchange pattern. Thus, the increased postprandial uptake of BCAA after two months of HFHS feeding could respond to the increased circulating concentration of BCAA already observed in obese hyperinsulinemic mini-pigs [5,52]. Indeed, high insulin levels have been shown to enhance BCAA uptake in the liver of obese rats [53] and promote further severe liver insulin resistance by the attenuation of Akt2 signaling via mTORC1- and mTORC2-dependent pathways [54].

## 5. Conclusions

Here, we showed that the unique exploration of the metabolome exchanges across the intestine and the liver allowed determining that obese mini-pigs not only adapted their splanchnic postprandial metabolism to the most abundant nutrients available, but also that hepatic metabolism was reorganized to maintain whole body glucose homeostasis and avoid the drift from insulin resistance onset into prediabetes. Thus, the splanchnic area remained flexible enough to adapt some aspects of the mainly glucose related metabolism, while other aspects (lipid handling, glycine, and creatine metabolisms) started to indicate the limit of adaptive capacities, which could eventually lead to overt prediabetes. On the other hand, metabolites related to lipid handling and energy metabolism showed a blunted postprandial response in the obese animals across organs, reflecting a loss of flexibility in response to the HFHF meal challenge in unsuspected metabolic pathways (paradoxical glycogen synthesis, formic acid use related to purine metabolism, etc.). We also showed that the proportion of the metabolome able to discriminate healthy from obese animals was greater after the meal than during the static homeostasis balance (fasting), particularly at the hepatic level. Finally, the specific postprandial changes of some of the metabolites discussed here (lactate, ethanolamine, glutamate, propionate, acetate, etc.) were particularity well correlated with the healthy outcomes (HOMA-IR or weight gain), and could constitute a postprandial signature of insulin resistance and obesity onset.

## Figures and Tables

**Table 1:** Percentage of induction of metabolites across the liver.

		Postprandial Time (Minutes)				
		0	60	180	330	510
**Lysine**	Day 0	8.97 ± 4.10	5.81 ± 1.67 *	2.08 ± 1.48	4.13 ± 0.86 *	6.26 ± 1.51 *
	Day 60	3.88 ± 3.35	6.57 ± 0.48 *	4.02 ± 2.28	7.68 ± 1.24 *	8.91 ± 1.45 *
**Threonine**	Day 0	−0.63 ± 3.73	2.61 ± 1.78	−2.44 ± 0.96	−4.69 ± 3.16	−0.36 ± 1.30
	Day 60	5.07 ± 3.66	5.21 ± 1.98	0.62 ± 1.76	3.39 ± 1.60	3.76 ± 4.54
**Citrate**	Day 0	30.87 ± 21.17	12.45 ± 1.52 *	8.66 ± 6.59	0.86 ± 10.14	17.66 ± 5.89 *
	Day 60	7.48 ± 7.95	1.74 ± 0.77	4.63 ± 11.26	7.53 ± 1.39 *	10.62 ± 7.47
**Isoleucine**	Day 0	4.97 ± 3.38	10.94 ± 2.26 *	26.12 ± 10.22 *	19.64 ± 9.22	21.38 ± 7.98 *
	Day 60	26.38 ± 22.41	3.10 ± 1.99	−3.29 ± 10.12	5.19 ± 2.52	6.30 ± 4.31
**Proline**	Day 0	15.17 ± 10.74	2.59 ± 3.13	2.10 ± 3.54	1.18 ± 1.72	0.06 ± 1.54
	Day 60	0.76 ± 1.02	0.59 ± 0.87	3.05 ± 1.46	3.65 ± 2.25	8.80 ± 1.98 *
**Acetate**	Day 0	1.06 ± 10.49	−1.68 ± 2.30	1.17 ± 0.80 *	−16.05 ± 4.62	−13.71 ± 5.11
	Day 60	4.92 ± 10.97	5.18 ± 8.58	−1.40 ± 11.18	3.55 ± 11.85	21.64 ± 22.29
**Tryptophan**	Day 0	−12.34 ± 7.34	−9.32 ± 7.77	−3.51 ± 7.79	−9.03 ± 5.57	6.81 ± 6.89
	Day 60	0.65 ± 9.26	5.50 ± 5.19	−0.91 ± 9.36	−5.34 ± 4.20	−16.44 ± 9.22
**Creatine**	Day 0	−0.94 ± 2.83	2.15 ± 3.22	−5.08 ± 1.21 *	2.37 ± 2.46	2.10 ± 1.91
	Day 60	−4.79 ± 7.77	2.53 ± 2.36	−5.16 ± 2.49	−2.75 ± 2.52	−5.53 ± 3.65
**Betaine**	Day 0	11.87 ± 2.11 *	15.29 ± 4.93 *	5.94 ± 2.83	17.69 ± 8.61	11.30 ± 2.32 *
	Day 60	15.79 ± 4.41 *	14.92 ± 2.32 *	10.70 ± 1.94 *	16.54 ± 3.10 *	12.88 ± 2.43 *
**Phosphocholine**	Day 0	−2.29 ± 4.76	3.74 ± 4.53	−7.12 ± 2.74	−2.58 ± 2.90	4.75 ± 3.29
	Day 60	5.87 ± 4.86	3.51 ± 2.78	−7.04 ± 4.61	−0.06 ± 3.08	−2.58 ± 4.65
**Alanine**	Day 0	51.82 ± 15.81 *	26.21 ± 4.81 *	25.36 ± 7.37 *	20.55 ± 0.97 *	24.69 ± 1.24 *
	Day 60	23.52 ± 7.30 *	20.34 ± 2.21 *	26.50 ± 3.85 *	28.78 ± 4.14 *	30.99 ± 3.74 *
**Asparagine**	Day 0	18.39 ± 9.83	5.54 ± 3.80	6.01 ± 3.38	3.54 ± 4.07	0.84 ± 3.13
	Day 60	−0.15 ± 2.64	2.09 ± 1.82	3.17 ± 1.09 *	4.72 ± 2.07	10.95 ± 7.37
**Methionine**	Day 0	12.27 ± 6.82	8.98 ± 1.52 *	4.90 ± 2.30	33.92 ± 4.22 *	36.07 ± 4.86 *
	Day 60	−2.62 ± 3.97	4.45 ± 0.60 *	4.97 ± 2.48	3.28 ± 2.54	6.90 ± 3.38
**Lipids**	Day 0	−0.46 ± 2.30	0.44 ± 0.61	0.44 ± 1.65	−0.91 ± 0.81	−2.75 ± 1.25
	Day 60	−0.80 ± 2.53	0.23 ± 1.79	0.81 ± 3.64	−1.00 ± 1.89	−1.37 ± 1.20
**Glycero-Phosphocholine**	Day 0	1.59 ± 3.92	4.11 ± 3.07	0.61 ± 1.54	4.12 ± 2.06	5.68 ± 1.37 *
	Day 60	4.14 ± 2.65	4.19 ± 2.52	−3.27 ± 2.14	0.50 ± 2.59	−2.16 ± 3.37
**Glutamine**	Day 0	19.13 ± 9.29 *	10.06 ± 3.71	11.42 ± 3.90 *	13.83 ± 3.56 *	17.78 ± 4.40 *
	Day 60	8.75 ± 6.11	12.56 ± 0.51 *	16.02 ± 5.74 *	20.83 ± 0.72 *	23.92 ± 5.11
**Valine**	Day 0	−9.98 ± 13.40	−6.63 ± 2.56	−19.59 ± 6.02 *	−10.05 ± 3.39 *	−11.89 ± 5.61
	Day 60	−9.99 ± 8.82	4.06 ± 1.81	9.75 ± 8.69	5.08 ± 2.03	5.76 ± 2.78
**Leucine**	Day 0	−4.25 ± 7.91	−1.69 ± 1.16	−6.50 ± 2.69	−2.97 ± 1.41	−3.25 ± 2.54
	Day 60	−4.04 ± 5.91	3.25 ± 0.95 *	3.88 ± 3.77	3.67 ± 1.3 *	2.84 ± 2.02
**Tyrosine**	Day 0	15.49 ± 4.37 *	8.92 ± 2.96 *	9.83 ± 3.76 *	7.55 ± 8.06	16.10 ± 3.99 *
	Day 60	8.91 ± 7.53	17.21 ± 3.49 *	20.15 ± 3.58 *	22.39 ± 2.77 *	20.90 ± 4.00 *
**Phenylalanine**	Day 0	12.11 ± 8.13	12.45 ± 4.40 *	8.16 ± 2.17 *	14.17 ± 3.47 *	15.69 ± 3.86 *
	Day 60	−24.07 ± 11.72	20.06 ± 3.20 *	15.18 ± 2.65 *	20.22 ± 1.80 *	12.62 ± 6.05
**Glucose**	Day 0	−6.91 ± 1.38 *	−6.28 ± 1.75 *	−4.48 ± 0.27 *	−3.69 ± 1.90	−2.19 ± 1.40
	Day 60	−5.82 ± 2.57	−5.70 ± 2.10	−4.80 ± 3.54	−5.36 ± 1.93	−4.87 ± 2.19

* significant (*p* < 0.05) exchange across the liver. negative values mean release and positive values mean uptake of a given metabolite.

**Table 2:** Percentage of induction of metabolites across the intestine.

		Postprandial Time (Minutes)				
		0	60	180	330	510
**Lysine**	Day 0	1.08 ± 1.22	1.79 ± 1.36	−2.42 ± 3.24	4.62 ± 1.74t	−1.32 ± 2.1
	Day 60	−1.09 ± 3.82	0.96 ± 1.38	−3.12 ± 1.73	−2.34 ± 1.16	−6.81 ± 2.09 *
**Threonine**	Day 0	10.53 ± 1.88 *	16.22 ± 1.85 *	8.56 ± 3.52t	20.03 ± 4.32 *	8.93 ± 5.06
	Day 60	5.97 ± 3.11	16.57 ± 4.02 *	11.55 ± 0.94 *	12.5 ± 2.32 *	4.40 ± 2.79
**Citrate**	Day 0	4.12 ± 2.64	0.83 ± 3.73	−9.27 ± 10.05	42.08 ± 27.92	2.13 ± 10.86
	Day 60	5.51 ± 2.66	9.65 ± 4.54t	2.18 ± 3.58	10.26 ± 6.4	−3.52 ± 6.86
**Isoleucine**	Day 0	18.41 ± 6.24 *	0.16 ± 7.15	2.65 ± 7.3	18.88 ± 6.04 *	2.84 ± 8.25
	Day 60	−4.48 ± 4.20	2.95 ± 2.58	1.49 ± 10.46	−10.25 ± 3.84t	−9.55 ± 7.47
**Valine**	Day 0	−8.30 ± 3.03t	14.95 ± 8.59	1.58 ± 5.99	−23.38 ± 11.61	−1.88 ± 11.28
	Day 60	6.20 ± 6.84	0.03 ± 1.20	−1.47 ± 5.02	5.81 ± 3.93	6.52 ± 8.09
**Leucine**	Day 0	−2.73 ± 1.57	10.95 ± 3.66 *	3.18 ± 1.67	−5.73 ± 3.12	−1.81 ± 4.28
	Day 60	2.61 ± 4.83	3.28 ± 1.14 *	−1.61 ± 1.99	1.79 ± 2.08	1.21 ± 3.27
**BCAA**	Day 0	−1.44 ± 1.22	9.34 ± 2.61 *	2.35 ± 0.90t	−4.60 ± 3.16	−1.44 ± 3.41
	Day 60	2.03 ± 4.97	2.48 ± 1.11t	−2.16 ± 1.59	0.44 ± 1.61	−0.13 ± 2.29
**Tryptophan**	Day 0	8.37 ± 5.91	12.87 ± 10.36	18.66 ± 7.73	26.83 ± 5.00t	16.55 ± 15.69 *
	Day 60	12.35 ± 1.85	26.8 ± 6.19	30.38 ± 12.89	12.02 ± 9.50	32.67 ± 11.17
**Betaine**	Day 0	1.31 ± 1.74	19.29 ± 2.68 *	16.22 ± 2.58 *	9.60 ± 4.30	4.66 ± 2.15
	Day 60	2.05 ± 2.62	23.74 ± 2.22 *	14.25 ± 1.06 *	10.15 ± 6.25	4.53 ± 4.29
**Phosphocholine**	Day 0	9.27 ± 1.87 *	16.01 ± 3.32 *	24.78 ± 7.04 *	39.95 ± 9.35 *	21.66 ± 7.24 *
	Day 60	8.00 ± 3.16t	34.4 ± 8.98 *	22.05 ± 6.45 *	17.73 ± 4.78 *	10.11 ± 6.58
**Alanine**	Day 0	−6.74 ± 1.81 *	−13.2 ± 0.93 *	−15.79 ± 2.1 *	−10.59 ± 2.01 *	−12.95 ± 1.59 *
	Day 60	−7.71 ± 4.07	−12.89 ± 0.65 *	−16.04 ± 2.00 *	−15.73 ± 2.17 *	−17.95 ± 2.63 *
**Asparagine**	Day 0	6.64 ± 4.36	6.73 ± 3.8	−10.33 ± 6.11	7.79 ± 6.85	−3.01 ± 5.40
	Day 60	5.73 ± 4.10	−1.89 ± 1.79	−8.88 ± 2.23 *	0.47 ± 1.90	−6.5 ± 3.73
**Formic**	Day 0	−31.91 ± 3.80 *	−26.38 ± 1.81 *	−25.4 ± 5.39 *	−29.63 ± 0.98 *	−21.06 ± 2.61 *
	Day 60	−17.67 ± 10.34	−33.63 ± 3.61 *	−22.35 ± 7.93 *	−23.58 ± 8.58 *	−26.17 ± 5.52 *
**Lipids**	Day 0	1.38 ± 1.95	21.6 ± 2.97 *	16.28 ± 3.19 *	9.45 ± 2.80 *	9.5 ± 2.38 *
	Day 60	7.44 ± 3.32t	21.71 ± 2.92 *	17.36 ± 2.53 *	16.06 ± 1.47 *	12.12 ± 2.5 *
**Methionine**	Day 0	7.15 ± 2.66 *	8.06 ± 1.50 *	−3.58 ± 3.87	16.68 ± 8.97 *	8.38 ± 6.08
	Day 60	15.06 ± 3.60 *	5.84 ± 2.85	−0.45 ± 1.28	10.42 ± 2.62 *	3.99 ± 3.63
**Glutamate**	Day 0	8.49 ± 0.54 *	9.56 ± 1.54 *	1.15 ± 3.04	8.15 ± 2.93 *	4.37 ± 3.74
	Day 60	9.99 ± 4.23t	9.32 ± 2.97 *	0.65 ± 2.56	4.25 ± 0.89 *	1.09 ± 1.98
**Lactate**	Day 0	15.06 ± 8.44	7.48 ± 9.83	−27.27 ± 10.61t	−11.56 ± 10.07	−30.78 ± 14.73
	Day 60	−1.53 ± 8.96	−16.06 ± 12.96	−4.54 ± 14.18	10.54 ± 29.75	−24.19 ± 5.68 *
**Pyruvate**	Day 0	−2.38 ± 2.9	4.9 ± 6.06	−1.57 ± 3.73	0.69 ± 2.47	−5.27 ± 4.76
	Day 60	−0.85 ± 6.94	7.34 ± 8.25	7.85 ± 6.43	−1.06 ± 6.09	−0.43 ± 7.74
**Tyrosine**	Day 0	2.51 ± 3.64	1.46 ± 3.66	−4.91 ± 2.19	8.61 ± 8.00	−6.76 ± 3.88
	Day 60	8.65 ± 6.07	1.48 ± 4.57	−0.58 ± 6.02	0.49 ± 5.72	1.68 ± 6.97
**Histidine**	Day 0	0.89 ± 2.05	3.79 ± 2.88	−3.51 ± 2.46	3.37 ± 2.88	−6.62 ± 2.98t
	Day 60	0.00 ± 1.64	4.55 ± 2.35	−5.08 ± 2.25t	−2.41 ± 1.45	−5.50 ± 3.45
**Ethanolamine**	Day 0	−4.79 ± 2.86	5.05 ± 2.36t	−8.8 ± 3.18 *	−5.01 ± 1.07 *	−5.82 ± 2.72t
	Day 60	−1.14 ± 4.21	−1.82 ± 3.69	−8.8 ± 2.82 *	1.47 ± 4.16	−4.98 ± 3.69

*, significant (*p* < 0.05) exchange across the intestine. negative values mean release and positive values mean uptake of a given metabolite.

## Supplementary Materials

The following are available online at https://www.mdpi.com/2072-6643/12/8/2442/s1, Figure S1: Scores plot of multi level sparse partial least squares discriminant analysis model (*n* = 5) after OSC filter. Analyses were focussed on the T0, T60, T180, T330 and T510 min after the meal for each vessel (artery, hepatic vein, portal vein) and days (D0 and D60). Figure S2: Validation ROC curves for mlPLSDA models focussed on the T0, T60 and T180 min, for the three vessels artery, hepatic vein, portal vein) and on the two separate days (D0 and D60). Table S1. Postprandial response of metabolites across the liver. Table S2. Postprandial response of metabolites across the intestine. Table S3: Pearson correlations performed between the HOMA-IR or the BW and the AUC exchange values for each metabolite to identify metabolites that are release or take up in greater/smaller amount depending on the HOMA-IR or BW. Table S4: Pearson correlations performed between the HOMA-IR or the BW and the AUC exchange values for each metabolite to identify metabolites that are release or take up in greater/smaller amount depending on the HOMA-IR or BW. Table S5: Pearson correlations performed between the HOMA-IR or the BW and the AUC exchange values for each metabolite to identify metabolites that are release or take up in greater/smaller amount depending on the HOMA-IR or BW. Table S6: Pearson correlations performed between the HOMA-IR or the BW and the AUC exchange values for each metabolite to identify metabolites that are release or take up in greater/smaller amount depending on the HOMA-IR or BW.
