# Supplementary material for: Postprandial NMR-Based Metabolic Exchanges Reflect Impaired Phenotypic Flexibility across Splanchnic Organs in the Obese Yucatan Mini-Pig
Source: Nutrients. 2020 Aug 14;12(8):2442. doi: 10.3390/nu12082442 (PMC7468879; doi:10.3390/nu12082442)
Supplement: Supplementary file 1 [file nutrients-12-02442-s001.zip › Table S2 intestine.pdf]

Supplementary Table 2. Postprandial response of metabolites across the intestine.

		Total postprandial AUC	Maximum delta change from fasting
Lysine	*Day 0*	1.05 ± 1.39	-2.72 ± 3.88
	Day 60	-2.41 ± 1.11	-1.90 ± 5.40
Threonine	*Day 0*	13.49 ± 2.64*	5.28 ± 7.87
	Day 60	11.47 ± 0.96*	11.83 ± 4.56
Citrate	*Day 0*	7.11 ± 5.52	21.70 ± 35.15
	Day 60	5.63 ± 3.44	1.78 ± 10.68
Isoleucine	*Day 0*	8.12 ± 4.34	-25.75 ± 11.28
	Day 60	-4.44 ± 4.58#	10.53 ± 8.82
Valine	*Day 0*	-6.35 ± 5.28	2.13 ± 21.55
	Day 60	2.63 ± 2.29	-15.01 ± 9.31
Leucine	*Day 0*	0.35 ± 1.65	12.94 ± 6.22
	Day 60	0.99 ± 0.52	-4.74 ± 6.97
BCAA	*Day 0*	0.33 ± 1.48	1.56 ± 6.99
	Day 60	0.08 ± 0.33	-4.65 ± 6.40
Tryptophan	*Day 0*	16.91 ± 2.29*	19.19 ± 19.04
	Day 60	21.09 ± 3.58*$	41.21 ± 7.97
Betaine	*Day 0*	10.18 ± 1.89*$	21.46 ± 2.04
	Day 60	11.72 ± 2.91*$	25.69 ± 2.42
Phosphocholine	*Day 0*	25.63 ± 4.86*	28.46 ± 12.71
	Day 60	19.27 ± 2.44*$	37.69 ± 7.56
Alanine	*Day 0*	-12.77 ± 0.95*$	-9.63 ± 3.67
	Day 60	-15.35 ± 1.32*	-6.37 ± 3.53
Asparagine	*Day 0*	-0.35 ± 2.82	-19.68 ± 9.61
	Day 60	-3.37 ± 1.05*	-15.12 ± 7.05
Formic	*Day 0*	-27.17 ± 1.47*	10.44 ± 5.98
	Day 60	-26.25 ± 5.30*	-10.28 ± 19.10
Lipids	*Day 0*	12.57 ± 1.34*$	21.31 ± 2.64
	Day 60	16.07 ± 1.81*$	16.50 ± 0.87
Methionine	*Day 0*	9.55 ± 3.27*	8.83 ± 11.69
	Day 60	7.33 ± 2.61*	-13.33 ± 7.44
Glutamate	*Day 0*	5.70 ± 1.48*	-7.75 ± 3.92
	Day 60	4.13 ± 0.97*	-6.64 ± 6.55
Lactate	*Day 0*	-14.89 ± 5.97$	-59.06 ± 6.86
	Day 60	-11.49 ± 7.97	15.70 ± 35.75
Pyruvate	*Day 0*	-0.85 ± 2.86	3.61 ± 8.89
	Day 60	2.94 ± 4.29	17.43 ± 12.17
Tyrosine	*Day 0*	-0.04 ± 2.82	2.16 ± 13.31
	Day 60	1.24 ± 5.54$	-7.59 ± 4.25
Histidine	*Day 0*	-0.45 ± 1.61	-3.30 ± 6.85
	Day 60	-2.01 ± 1.18	0.98 ± 4.57
Ethanolamine	*Day 0*	-4.46 ± 1.08*	2.50 ± 7.48
	Day 60	-3.08 ± 1.91	-6.73 ± 8.90

*, significant ($p<0.05$) exchange across the intestine. For the AUC, negative values mean release and positive values means uptake of a given metabolite. For the delta change, negative values mean decrease, while positive values mean increase in the exchange of a given metabolite following the meal. $, significant different between the fasting and the integrated postprandial exchange ($p<0.05$). Comparison against D0: #, $p<0.05$.
