# Supplementary material for: Postprandial NMR-Based Metabolic Exchanges Reflect Impaired Phenotypic Flexibility across Splanchnic Organs in the Obese Yucatan Mini-Pig
Source: Nutrients. 2020 Aug 14;12(8):2442. doi: 10.3390/nu12082442 (PMC7468879; doi:10.3390/nu12082442)
Supplement: Supplementary file 1 [file nutrients-12-02442-s001.zip › Suppl Figure 1.pdf]

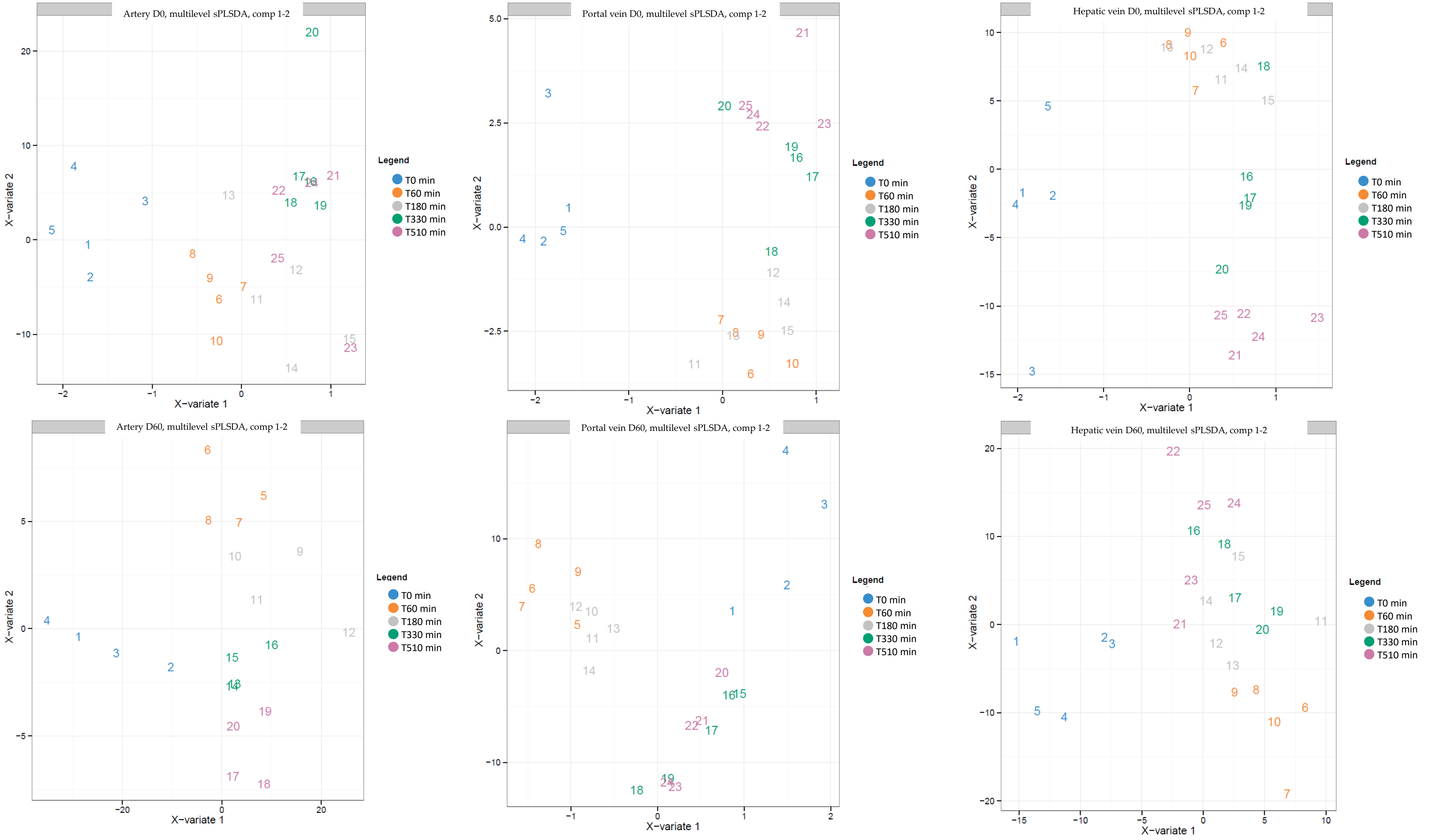

Supplementary Figure 1. Scores plot of multi-level sparse partial least squares discriminant analysis model (n=5) after OSC filter. Analyses were focussed on the T0, T60, T180, T330 and T510 min after the meal for each vessel (artery, hepatic vein, portal vein) and days (D0 and D60)
