# Supplementary material for: Postprandial NMR-Based Metabolic Exchanges Reflect Impaired Phenotypic Flexibility across Splanchnic Organs in the Obese Yucatan Mini-Pig
Source: Nutrients. 2020 Aug 14;12(8):2442. doi: 10.3390/nu12082442 (PMC7468879; doi:10.3390/nu12082442)
Supplement: Supplementary file 1 [file nutrients-12-02442-s001.zip › Suppl Figure 2.pdf]

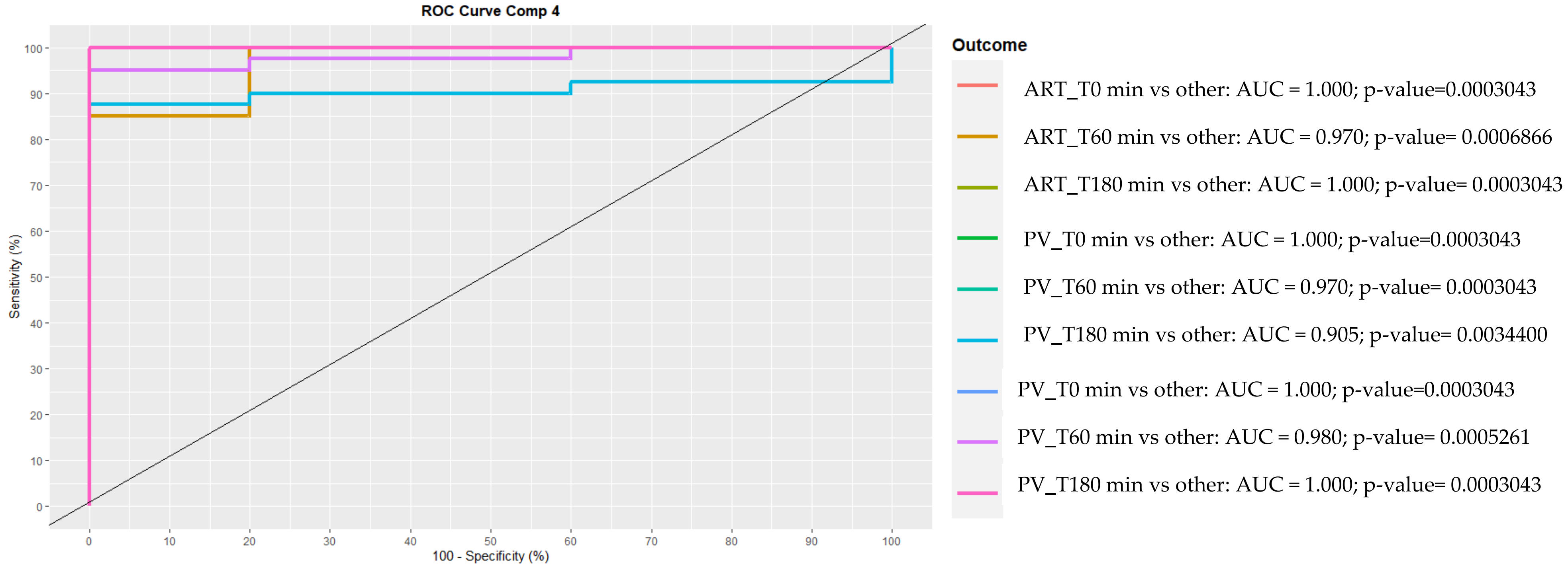

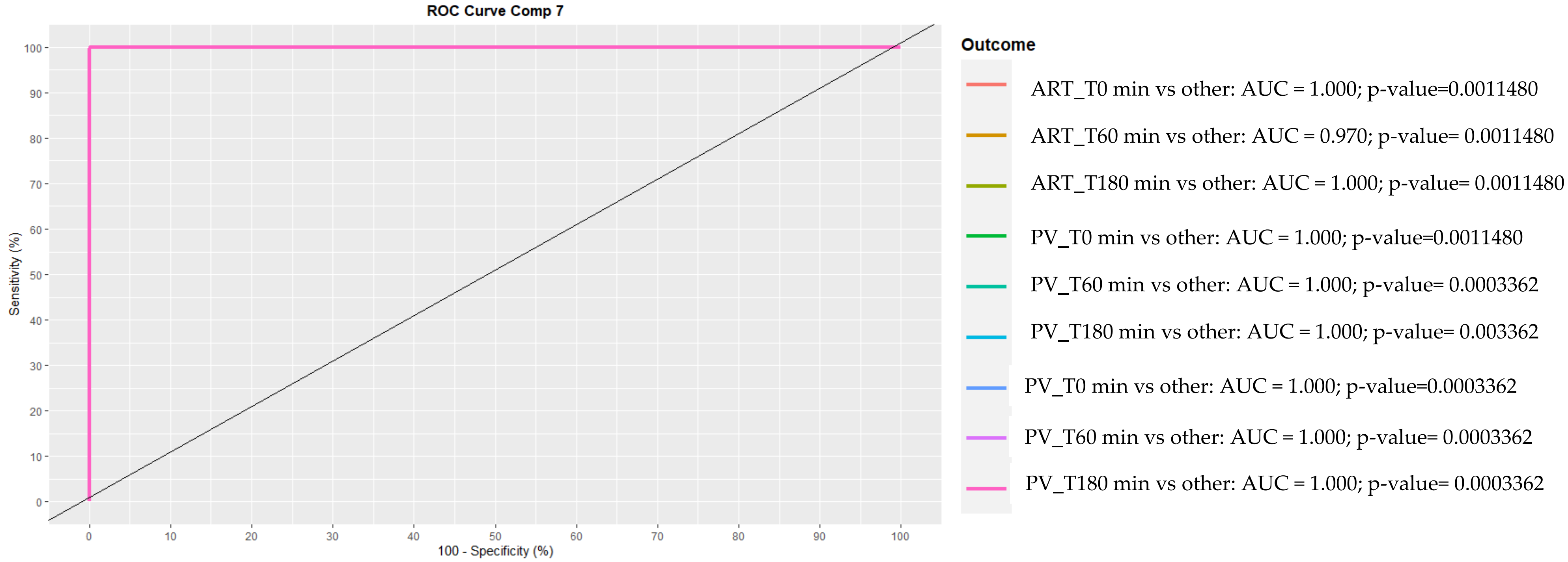

Supplementary Figure 2. Validation ROC curves for mlPLSDA models focussed on the T0, T60 and T180 min, for the three vessels (artery, hepatic vein, portal vein) and on the two separate days (D0 and D60)
