# Supplementary material for: Postprandial NMR-Based Metabolic Exchanges Reflect Impaired Phenotypic Flexibility across Splanchnic Organs in the Obese Yucatan Mini-Pig
Source: Nutrients. 2020 Aug 14;12(8):2442. doi: 10.3390/nu12082442 (PMC7468879; doi:10.3390/nu12082442)
Supplement: Supplementary file 1 [file nutrients-12-02442-s001.zip › Supplementary Table S3-S6.pdf]

Supplementary Table S3. Pearson correlations performed between the HOMA-IR or the BW and the AUC exchange values for each metabolite to identify metabolites that are release or take up in greater/smaller amount depending on the HOMA-IR or BW.

HOMA-IR *vs* Liver metabolites AUC		
	correlation coefficient	p-value
Citrate	-0.39	0.2640
Glycerophosphocholine	-0.35	0.3276
Acetate	-0.10	0.7798
Betaine	0.00	0.9910
Alanine	0.01	0.9837
Methionine	0.05	0.8967
Gluconeogenic AA	0.10	0.7797
Choline	0.13	0.7234
Phenlyalanine	0.14	0.7069
Asparragine	0.23	0.5222
Glycine	0.24	0.5038
Lysine	0.25	0.4779
Glutamine	0.33	0.3542
Glucose	0.35	0.3238
Threonine	0.38	0.2829
Phosphocholine	0.40	0.2572
Tryptophan	0.41	0.2453
Histidine	0.46	0.1762
Succinate	0.49	0.1550
Creatine	0.49	0.1520
Formic acid	0.52	0.1234
Lipids	0.53	0.1135
Propionate	0.54	0.1108
Tyrosine	0.59	0.0734
Glutamate	0.65	0.0439
Proline	0.69	0.0269
Ethanolamine	0.76	0.0100
Lactate	0.90	0.0004

Supplementary Table S4. Pearson correlations performed between the HOMA-IR or the BW and the AUC exchange values for each metabolite to identify metabolites that are release or take up in greater/smaller amount depending on the HOMA-IR or BW.

BW *vs* Liver metabolites AUC		
	correlation coefficient	p-value
Citrate	-0.34	0.333
Choline	-0.11	0.762
Glycerophosphocholine	-0.09	0.812
Betaine	0.18	0.612
Glucose	0.20	0.586
Methionine	0.23	0.527
Phenlyalanine	0.30	0.402
Creatine	0.32	0.373
Tyrosine	0.32	0.370
Threonine	0.34	0.341
Gluconeogenic AA	0.40	0.248
Acetate	0.42	0.227
Asparragine	0.42	0.224
Succinate	0.44	0.198
Glutamine	0.45	0.189
Alanine	0.50	0.137
Phosphocholine	0.51	0.136
Tryptophan	0.55	0.102
Glycine	0.59	0.073
Lactate	0.61	0.064
Formic acid	0.63	0.046
Lysine	0.64	0.045
Histidine	0.67	0.033
Lipids	0.70	0.024
Proline	0.71	0.022
Propionate	0.78	0.007
Glutamate	0.79	0.007
Ethanolamine	0.85	0.002

Supplementary Table S5. Pearson correlations performed between the HOMA-IR or the BW and the AUC exchange values for each metabolite to identify metabolites that are release or take up in greater/smaller amount depending on the HOMA-IR or BW.

HOMA-IR *vs* Intestine metabolites AUC		
	correlation coefficient	p-value
Asparragine	-0.58	0.0794
Citrate	-0.53	0.1188
Phosphocholine	-0.41	0.2334
Histidine	-0.35	0.3271
Glutamine	-0.34	0.3319
Glutamate	-0.34	0.3371
Glycerophosphocholine	-0.32	0.3724
Threonine	-0.29	0.4220
Lysine	-0.21	0.5689
Methionine	-0.10	0.7769
Formic acid	-0.06	0.8723
Phenlyalanine	-0.03	0.9360
Lactate	0.06	0.8714
Choline	0.10	0.7821
Betaine	0.26	0.4691
Alanine	0.28	0.4338
Lipids	0.29	0.4212
Proline	0.29	0.4088
Tyrosine	0.30	0.4076
Gluconeogenic AA	0.31	0.3802
Succinate	0.36	0.3106
Ethanolamine	0.36	0.3067
Glycine	0.41	0.2349
Tryptophan	0.53	0.1119
Acetate	0.57	0.0826
Glucose	0.63	0.0490
Creatine	0.66	0.0371
Propionate	0.69	0.0281

Supplementary Table S6. Pearson correlations performed between the HOMA-IR or the BW and the AUC exchange values for each metabolite to identify metabolites that are release or take up in greater/smaller amount depending on the HOMA-IR or BW.

BW *vs* Intestine metabolites AUC		
	correlation coefficient	p-value
BCAA AUC-I	-0.60	0.0479
Glutamine	-0.51	0.1281
Citrate	-0.40	0.2542
Lactate	-0.28	0.4352
Phosphocholine	-0.24	0.5052
Glycerophosphocholine	-0.22	0.5477
Glutamate	-0.19	0.6084
Formic acid	-0.10	0.7768
Methionine	-0.07	0.8378
Threonine	-0.07	0.8529
Histidine	-0.06	0.8775
Tyrosine	0.05	0.8917
Choline	0.05	0.8806
Succinate	0.08	0.8358
Asparragine	0.09	0.8143
Betaine	0.10	0.7882
Ethanolamine	0.13	0.7124
Phenlyalanine	0.18	0.6100
Proline	0.23	0.5312
Lysine	0.31	0.3792
Creatine	0.41	0.2358
Tryptophan	0.45	0.1954
Lipids	0.53	0.1150
Alanine	0.61	0.0625
Gluconeogenic AA	0.65	0.0416
Acetate	0.77	0.0089
Propionate	0.79	0.0063
Glucose	0.81	0.0042
Glycine	0.90	0.0003
