# Supplementary material for: Postprandial NMR-Based Metabolic Exchanges Reflect Impaired Phenotypic Flexibility across Splanchnic Organs in the Obese Yucatan Mini-Pig
Source: Nutrients. 2020 Aug 14;12(8):2442. doi: 10.3390/nu12082442 (PMC7468879; doi:10.3390/nu12082442)
Supplement: Supplementary file 1 [file nutrients-12-02442-s001.zip › Table S1 liver.pdf]

Supplementary Table 1. Postprandial response of metabolites across the liver.

		Total postprandial AUC	Maximum delta change from fasting
Lysine	*Day 0*	4.47 ± 0.27*	-6.02 ± 5.53
	Day 60	6.43 ± 0.96*	2.90 ± 5.68
Threonine	*Day 0*	-1.82 ± 1.10	-3.31 ± 6.47
	Day 60	3.02 ± 1.40	-4.77 ± 5.26
Citrate	*Day 0*	8.59 ± 4.45	-26.02 ± 23.39
	Day 60	4.71 ± 2.31	7.37 ± 19.32
Isoleucine	*Day 0*	18.26 ± 4.23*	31.13 ± 12.18
	Day 60	2.36 ± 3.70#	-28.37 ± 26.22
Proline	*Day 0*	1.85 ± 1.04	-19.05 ± 10.17
	Day 60	3.42 ± 1.03*$	7.86 ± 3.56
Acetate	*Day 0*	-8.80 ± 2.77	-10.32 ± 14.96
	Day 60	1.44 ± 6.98	1.68 ± 34.98
Tryptophan	*Day 0*	-6.33 ± 1.95*	11.46 ± 15.22
	Day 60	-5.22 ± 3.78	-4.42 ± 18.76
Creatine	*Day 0*	0.03 ± 0.78	2.70 ± 6.23
	Day 60	-3.21 ± 1.43	-0.55 ± 11.17
Betaine	*Day 0*	13.15 ± 3.11*	3.48 ± 11.04
	Day 60	14.26 ± 1.15*	-1.23 ± 7.60
Phosphocholine	*Day 0*	-1.63 ± 0.89	3.52 ± 9.56
	Day 60	-1.91 ± 0.91	-14.12 ± 9.29
Alanine	*Day 0*	24.04 ± 2.66*	-36.96 ± 14.06
	Day 60	26.21 ± 2.74*	2.45 ± 10.29
Asparagine	*Day 0*	4.53 ± 1.98	-17.34 ± 11.51
	Day 60	4.15 ± 1.32*	13.02 ± 9.41
Methionine	*Day 0*	0.27 ± 2.80	-23.02 ± 8.09
	Day 60	4.63 ± 1.96	12.49 ± 6.21#
Lipids	*Day 0*	-0.71 ± 0.46	-1.52 ± 3.43
	Day 60	-0.24 ± 1.91	0.66 ± 4.50
Glycero-phosphocholine	*Day 0*	3.31 ± 0.26	3.18 ± 4.85
	Day 60	-0.26 ± 1.32	-9.97 ± 5.30
Glutamine	*Day 0*	13.67 ± 2.77*	-0.62 ± 13.78
	Day 60	16.99 ± 1.30*	15.73 ± 12.91
Valine	*Day 0*	-13.61 ± 3.60*	-8.58 ± 17.11
	Day 60	4.90 ± 2.42#	21.34 ± 14.22
Leucine	*Day 0*	-4.06 ± 1.47	2.41 ± 9.12
	Day 60	2.92 ± 1.27#	7.13 ± 8.87
Tyrosine	*Day 0*	9.88 ± 3.65*	-10.76 ± 10.24
	Day 60	19.64 ± 3.16*,#	12.78 ± 7.76
Phenylalanine	*Day 0*	12.22 ± 2.30*	2.86 ± 11.29
	Day 60	12.54 ± 3.06*$	50.43 ± 11.23
Glucose	*Day 0*	-4.24 ± 0.74*	3.92 ± 3.50
	Day 60	-5.17 ± 2.14	1.01 ± 5.14

*, significant (p<0.05) exchange across the liver. For the AUC, negative values mean release and positive values means uptake of a given metabolite. For the delta change, negative values mean decrease, while positive values mean increase in the exchange of a given metabolite following the meal. $, significant different between the fasting and the integrated postprandial exchange (p<0.05). Comparison against D0: #, p<0.05.
